# Retinal Hemorrhage Caused by Lithemia

**Published:** 1897-02

**Authors:** A. Bethune Patterson

**Affiliations:** Atlanta, Ga.


### RETINAL HEMORRHAGE CAUSED BY LITHEMIA.

By A. BETHUNE PATTERSON, M. D., Atlanta, Ga.

In looking up the etiology of retinal hemorrhages in some of our text-books, I find quite a number of causes are given—among them gout and rheumatism, and I believe it is the custom of many eye surgeons to exclude these as probable causes of the retinal affection where there have not been distinct attacks of the general disease. The etiology of gout and rheumatism is by no means settled, some writers seeming to think that lithemia is the cause of these pathological conditions that vary so widely in their clinical history. My own experience would lead me to conclude that the accumulation of uric acid in the blood is not sufficient to bring about an attack of gout, or rheumatism. I find lithemia a very common condition, while gout and rheumatism are comparatively rare, and then, too, the remedies that so soon correct lithemia have slight retarding influence upon gout and rheumatism. The almost invariable digestive disorders which accompany these diseases, would naturally direct one's attention to the alimentary tract as the probable seat of special forms of ferments, which are responsible for these distinct pathological conditions. Murchison, it was, who first gave the name lithemia to the abnormal retention in the blood of uric acid; since his studies, considerable attention has been given to the subject by pathologists. About forty different complaints have been traced to the accumulation of uric acid in the blood.

The object of this paper is to attract the attention of the ophthalmologist to lithemia as a frequent cause of retinal hemorrhages. My conclusions are based upon a number of such cases. I can safely state that in arriving at a diagnosis by exclusion, every condition that favored the accident, was critically considered. It has been my habit for years to make a chemical and microscopic examination of the urine of patients. About three years ago, my attention was first attracted to uric acid salts in the urine of patients suffering with conjunctivitis, asthenopia, retinal hemorrhages, etc. I have the record of over thirty cases of asthenopia which continued after errors of refraction had been corrected and heterophoria excluded, who were relieved by remedies which corrected the excess of uric acid.

While serving as clinical assistant in The Royal London Ophthalmic Hospital, I noticed Drs. Nettleship, Dang and the other surgeons would often make a diagnosis of gouty, or rheumatic conjunctivitis, etc., when patients were subject to attacks of these troubles. On referring to notes taken at the clinics of De Wecker, Galezowski and Panas, of Paris, I find inquiry was frequently made of patients, as to attacks of gout and rheumatism. The cases upon which I have based my conclusions, with the exception of two or three, had never suffered with rheumatism, or gout. The following three cases which I give are illustrative of retinal hemorrhages caused from lithemia:

CASE I.—Mr. C. W. S., age 45; a lawyer. He had trouble with eyes for years; describes them as being "weak." About four or five years ago was treated by an oculist for conjunctivitis; glasses were also ordered. First came under my care in June, '95, suffering with pain in head and right eye, soreness in latter, also brow and cheek bone. It was during this attack that he discovered vision in right eye almost gone. Ophthalmoscopic examination showed there was a recent hemorrhage in region of macula, pilocarpine internally, and drops of atropine to keep the eye quiet. I lost sight of patient until February, '96, when he returned, complaining of pain in head and eye (left), soreness in brow and cheek bone. Tension increased with tenderness; complains of debility and lassitude with accompanying asthenopia; was unable to attend to office work; face pale, and anxious look; temperature normal; pulse 100 and tense; no organic lesion of heart; but evidence of hypertrophy; ophthalmoscope revealed fresh hemorrhage. Urine analysis; sp. grav. 1032, reaction acid; no albumin, or sugar, heavily charged with crystals of uric acid, and at times scalding. Never had gout, or rheumatism; has had several attacks of renal colic during the last five or six years, also several attacks of what was thought to be hepatic colic; up to these attacks had suffered with dyspepsia; has been quite free of the distressing symptom of late. Believing these symptoms to be due to lithemia, I placed the patient at once upon Alkalithia. Rochelle Salts to relieve the bowels and to drink freely of Bowden Lithia water. A month later reported his condition improved; has been free of pain in head and eye. At this writing is conducting the work at his office with comfort and with no history of recurrent hemorrhage and with improvement in health generally.

CASE II.—Miss. M. C., age 20. From childhood has suffered with bilious attacks, constipation and general disturbance of digestive organs; mental depression and irritability; pain in head and eyes, smarting and gritty sensation in lids; so great was the asthenopia that she was unable to continue at school notwithstanding the treatment she had received for conjunctivitis and the correction of half a dioptrie of hypermetropia. Saw her a few hours after the retinal hemorrhage occurred; stated that for several days she had been suffering with indigestion, languid and depressed; headache and inability to use eyes for near work. Temperature normal, pulse 110. No organic lesion. Urine test, sp. grav. 1030. No albumin, or sugar, acid reaction, high colored, scant and burning, highly charged with crystals of uric acid; had a slight attack of rheumatism when a child. Ordered a purgative and a mixture of acetate potash, digitalis and salicylate soda and to drink freely of water. I have kept this case under observation now for over two years. There has been no return of hemorrhage and she is entirely relieved of asthenopia. She returns to her medicine whenever she feels symptoms of lithemic outbreak.

Case III.—C. A. Mc., age 45. For years has suffered with attacks of indigestion accompanied by constipation, loss of appetite, sour stomach; pain in head and eyes; loss of energy, accompanied by mental hebetude and despondency. History of eye, strain for near work; wears 2.50 which has proved satisfactory. Has never had gout or rheumatism. Urine frequently high-colored, scant and scalding. In February last during one of these attacks had an acute pain in left eye and at once discovered that he was almost blind in it. He consulted an oculist, who discovered a recent hemorrhage of retina. The patient was placed upon pilocarpine internally and atropine drops to keep the eye quiet. Five months later he came under my care with history of several hemorrhages. I found the eye under the influence of atropine; tension increased and ball tender; at times the pain in the eye was very severe. No light perception. Urine analysis, sp. grav. 1028; reaction acid. No trace of albumin or sugar; high-colored, scant, and burning; heavily charged with uric acid crystals. Atropine discontinued and eserine instilled, I think the tension was largely due to dilated iris encroaching upon the drainage tubes. Rochelle salts were ordered to be taken every morning before breakfast to keep the bowels in a relaxed condition. Alkalithia four times a day and to drink copiously of Bowden Lithia Water. He reported at the end of a week, very much improved; feels brighter and more cheerful, has had no pain in head or eye; tension nearly normal. At the end of second week eye still comfortable with no return of pain, the longest period of freedom from pain since first attack. Sp. grav. of urine 1022; reaction acid. No albumin, or sugar. Microscope showed a great diminution in uric acid.

Since writing the above, I have read in the New York *Medical Journal* an article by Thomas J. Mays, on the treatment of hemoptysis. The following quotation illustrates the degenerating influence of uric acid upon the capillaries of the lungs. He says: "Dr. Haig, in his work on uric acid, speaks of the important relationship which exists between uric acid in many cases of hemoptysis, and advocates the administration of the salicylates under such circumstances. At first sight, this connection may seem rather vague, but the good effect which follows such a line of treatment makes it altogether obvious. There is no doubt in my mind that uric acid sustains some causative relation to pulmonary blood spitting, for I have seen many striking examples in which the sodium salicylates exercised greater hemostatic properties than anything else which was administered. The following is a typical example of this influence." After reporting the case, he continues: "This was my first case of hemoptysis in which I was brought fully to realize that rheumatism might become one of its important complications, and serve to point out the proper line of giving relief, and this fortunate realization I ascribe wholly to my familiarity with the work of Dr. Haig. I have seen the same condition since, in other cases, and met its therapeutic indications by administering the salicylate of sodium in proper doses. Dr. Haig also advises the giving of mercury iodide, for the same purpose, where there is an excess of uric acid in the blood, as indicated by crystals of urine acid salts found in the urine by microscopic examination." I usually prescribe Alkalithia, and the drinking freely of Bowden Lithia Water. I find this sufficient in combination with saline laxatives, especially so in red and inflamed lids, and in many cases of chronic catarrh of the throat and middle ear.

